# High uptake of menstrual health information, products and analgesics within an integrated sexual reproductive health service for young people in Zimbabwe

**DOI:** 10.1186/s12978-024-01789-y

**Published:** 2024-04-22

**Authors:** Mandikudza Tembo, Victoria Simms, Helen A. Weiss, Tsitsi Bandason, Nicol Redzo, Leyla Larsson, Ethel Dauya, Tafadzwa Nzanza, Pauline Ishumael, Nancy Gweshe, Rangarirai Nyamwanza, Precious Ndlovu, Sarah Bernays, Chido Dziva Chikwari, Constancia Vimbayi Mavodza, Jenny Renju, Suzanna C. Francis, Rashida A. Ferrand, Constance Mackworth-Young

**Affiliations:** 1 MRC International Statistics & Epidemiology Group, London School of Hygiene and Tropical Medicine, London, UK; 2 The Health Research Unit Zimbabwe, Biomedical Research and Training Institute, Harare, Zimbabwe; 3 Department of Clinical Research, London School of Hygiene and Tropical Medicine, London, UK; 4 Department of Public Health, Environments and Society, Faculty of Public Health and Policy, London School of Hygiene and Tropical Medicine, London, UK; 5 Department of Global Health and Development, Faculty of Public Health and Policy, London School of Hygiene and Tropical Medicine, London, UK; 6 Division of Infectious Diseases and Tropical Medicine, Ludwig Maximilian University Hospital, Munich, Germany; 7 School of Public Health, University of Sydney, Sydney, NSW Australia

**Keywords:** Menstrual health, Integrated services, Adolescents, Menstruation, Community-based

## Abstract

**Background:**

Despite being integral to women’s well-being, achieving good menstrual health (MH) remains a challenge. This study examined MH services uptake (including information, analgesics, and a choice of MH products - the menstrual cup and reusable pads) and sustained use of MH products within an integrated sexual and reproductive health intervention for young people in Zimbabwe.

**Methods:**

This mixed-methods study was nested within a cluster randomised trial of integrated sexual and reproductive health services (CHIEDZA) for youth in three provinces (Harare, Mashonaland East, and Bulawayo). The study collected qualitative and quantitative data from 27,725 female clients aged 16–24 years, who accessed CHIEDZA from April 2019 – March 2022. Using a biometric (fingerprint recognition) identification system, known as SIMPRINTS, uptake of MH information, products, and analgesics and other services was tracked for each client. Descriptive statistics and logistic regression were used to investigate MH service uptake and product choice and use over time, and the factors associated with these outcomes. Thematic analysis of focus group discussions and interviews were used to further explore providers’ and participants’ experiences of the MH service and CHIEDZA intervention.

**Results:**

Overall, 36,991 clients accessed CHIEDZA of whom 27,725 (75%) were female. Almost all (*n* = 26,448; 95.4%) took up the MH service at least once: 25433 took up an MH product with the majority (23,346; 92.8%) choosing reusable pads. The uptake of cups varied across province with Bulawayo province having the highest uptake (13.4%). Clients aged 20–24 years old were more likely to choose cups than reusable pads compared with those aged 16–19 years (9.4% vs 6.0%; *p* < 0.001). Over the implementation period, 300/1819 (16.5%) of clients swapped from the menstrual cup to reusable pads and 83/23346 (0.4%) swapped from reusable pads to the menstrual cup. Provision of the MH service encouraged uptake of other important SRH services. Qualitative findings highlighted the provision of free integrated SRH and MH services that included a choice of MH products and analgesics in a youth-friendly environment were key to high uptake and overall female engagement with SRH services.

**Conclusions:**

High uptake demonstrates how the MH service provided much needed access to MH products and information. Integration of MH within an SRH intervention proved central to young women accessing other SRH services.

**Supplementary Information:**

The online version contains supplementary material available at 10.1186/s12978-024-01789-y.

## Plain English summary

Menstrual health (MH) is integral to women’s sexual and reproductive health and wellbeing. Yet it remains a challenge for many across the globe. In Zimbabwe, there is a need to improve MH as critical gaps remain in access to MH information, support, and products. This study investigated the uptake and use of an MH service (including information, pain medication, and a choice of menstrual products - the menstrual cup and reusable pads) within an integrated sexual and reproductive health intervention for young people in Zimbabwe called CHIEDZA. The study collected data from 27,725 female clients aged 16–24 years, who accessed CHIEDZA from April 2019 – March 2022. Interviews were used to further explore providers’ and clients’ experiences of CHIEDZA. Almost all female clients took up the MH service at least once. Of those who took up a menstrual product, most chose reusable pads over the cup. Menstrual product choice varied by age, with older versus younger clients being more likely to choose cups over reusable pads. Most clients were happy with their initial menstrual product choice. However, some clients swapped products. More clients swapped from the cup to reusable pads than vice versa. The provision of free integrated services that included a choice of menstrual products and pain medication in a youth-friendly environment were key to high uptake and overall female engagement with other important sexual and reproductive health services in CHIEDZA. Our study shows that MH is a key part of sexual and reproductive health service provision of young women.

## Introduction

Menstrual health (MH), defined as having access to the materials, facilities, and support to manage menstruation with privacy and dignity, is integral to women’s empowerment, reproductive health, and overall wellbeing [1, 2]. To effectively manage menstruation, women need to have access to affordable and effective menstrual products, pain management, education and support, and water, sanitation and hygiene (WASH) facilities [1, 3]. As well as lacking access to these, many women are also affected by both the shame and taboo around menstruation [2, 4, 5]. Lack of access to support and MH products can lead to embarrassment and isolation during menstruation, school drop-out, and transactional sex, which in turn increases the risk of early age at pregnancy and sexually transmitted infections [5–11].

With a growing understanding of the importance of MH, a variety of interventions have been implemented to mitigate the impact of inadequate MH [12]. As of 2022, these include community-based interventions [13], multicomponent interventions [14], and interventions that address the MH needs of those with disabilities [15]. However, most of these interventions have 1) framed MH as an isolated issue, 2) targeted school-going girls, and 3) focused on the provision of MH education or products, but rarely both [16].

MH is now widely accepted as a core component of sexual and reproductive health (SRH) [1, 17]. Menstruation is a key feature of women’s reproductive years thus making MH a natural entry point and acceptable pathway to understanding and addressing women’s reproductive needs [17, 18]. Integrating MH into SRH service provision can potentially be an effective and acceptable model to maximize use of healthcare resources, provide comprehensive care and increase engagement with critical health services in the community [19]. This is important as young women face many barriers when seeking healthcare and are often the most vulnerable to adverse reproductive health outcomes linked to risky sexual practices [20–22]. Despite this, there is limited research work to inform best practices and acceptable and sustainable approaches to integrated service provision approaches for women across the globe [18].

In Zimbabwe, most young women have limited access to appropriate MH support and other SRH services such as contraception and HIV testing [23–25]. Young women in low-income areas throughout Zimbabwe have had to improvise and use alternative materials such as old fabric or newspaper to manage their menstruation [26]. Using these alternatives poses high risk of discomfort, skin irritation and/or leakage. As a result, many end up missing school or work due to humiliation and stigma [26, 27]. Others also report missing work or school due a lack of access to MH-related pain management [28, 29]. Even with these MH-related issues and the global push for MH interventions in LMICs, studies specific to Zimbabwe remain limited [28]. Critical gaps remain in addressing the MH needs of women in Zimbabwe [30], despite efforts made by Government and developmental agencies to achieve the Sustainable Development Goals by 2030 [28, 31]. Apart from a small feasibility study conducted to investigate menstrual practices and perceptions around the use of the Duet (an insertable MH product) [23] and another study looking at the acceptability of menstrual cups [28], the limited literature on MH and/or MH and SRH integration in Zimbabwe highlights a key evidence gap in how best to improve young women’s health and overall well-being. It is critical that this research is conducted and used to advocate for evidence-driven policy-making that mainstreams MH and places MH at the center of SRH for girls and women in Zimbabwe.

The aim of this study was to investigate the uptake and usage patterns of MH services including analgesics, MH education and a choice of MH products (the menstrual cup or reusable pads) within a comprehensive integrated SRH service.

## Methods

### Study design and setting

This mixed-methods study was nested within a cluster-randomised trial (called CHIEDZA) aimed at investigating the impact of community-based integrated HIV and SRH services for young people in Zimbabwe aged 16–24 years on population-level HIV outcomes. (CHIEDZA trial registration number in clinical trials.gov: NCT03719521) [32]. The trial protocol has been described elsewhere [32] but in brief the CHIEDZA intervention included HIV testing and treatment with adherence support, as well as family planning, management of sexually transmitted infections (STIs), condoms, referrals for voluntary medical male circumcision (VMMC), counselling, and MH services (described below), provided free-of-cost in a youth-friendly environment. The intervention was delivered by a multidisciplinary team of trained healthcare providers comprising two youth workers, one counsellor, two nurses, and four community health workers (CHWs). CHIEDZA was conducted in three provinces in Zimbabwe (Harare, Bulawayo, and Mashonaland East) with eight clusters per province (total 24 clusters) randomised 4:4 to the intervention or to existing services (control arm) from April 2019 – March 2022. A cluster was defined as a geographically defined area with a community centre from where services were delivered.

Training of providers included comprehensive MH education that addressed puberty and body autonomy, harmful myths and taboos around menstruation, and correct use and maintenance of reusable MH products. Training materials used can be found elsewhere (www.chiedza.com). Providers were also given menstrual cups and reusable pads for their own use.

### Menstrual health services

The MH services available to all female CHIEDZA clients included two pairs of underwear, MH education and support, a simple paper-based period-tracking diary, a bar of soap, a choice of analgesics (12 tablets of paracetamol or ibuprofen available on a monthly basis), and a choice between either a menstrual cup (the Butterfly Cup that can used for up to 10 years) (www.vivalily.com) or a four-pack of reusable pads which included three normal sized pads and one night-time pad (AFRIpads™ that can be used for up to 2 years) (www.afripads.com). The MH service also included trained menstrual cup ambassadors on site for menstrual cup sensitization and user support over time. All female CHIEDZA clients also had the option to swap their original MH product choice for another after a minimum 3 months of usage.

### Quantitative data collection methods

CHIEDZA was a walk-in, once-a-week community based service open to all young people living in the intervention clusters. Every client attendance to CHIEDZA was captured using a biometric system (SIMPRINTS, Cambridge, UK) [33]. At first visit, a client provided a registration fingerprint which was converted into a unique identifier. Age, sex, and type of service taken up at each visit was documented for each client, linked to their unique ID. No sociodemographic or clinical information was collected. Clients gave verbal consent for services as need for verbal consent was waived. At subsequent visits, clients provided fingerprints which enabled tracking of services taken up by clients over time.

Data was collected using electronic tablets. All female clients that attended CHIEDZA were eligible to take up the MH service. Uptake of MH information, product, and type and/or analgesics was recorded. If a client’s choice of MH product or analgesics was unavailable, this was recorded as a “stock-out” occurrence and clients were advised on when to return to pick up their preferred product and/or analgesics.

### Quantitative data analysis

MH service uptake across all the 12 intervention clusters was calculated as the proportion of women who took up MH services of those who attended CHIEDZA. Logistic regression with a random effect for cluster was used to estimate the association of uptake of MH services and MH product choice with province and age group. Data were analysed using Stata version 17 (StataCorp, Texas, USA).

### Qualitative data collection methods

We conducted non-participant observations of CHIEDZA sites, and in-depth interviews (IDIs), and focus group discussions (FGDs) with CHIEDZA service providers and clients. Data was collected by three trained qualitative researchers (CVM, RN, PN) who were independent of the implementation team. All interviews were conducted in Shona, English, or Ndebele (as preferred by the participants), lasted between 20 and 100 minutes, and were conducted in-person or by phone (when COVID-19 restrictions prevented in-person engagement).

Semi-structured topic guides were iteratively informed by preliminary findings from previously collected data and discussions with the qualitative team. The interviews explored participants’ perspectives on CHIEDZA and the overall acceptability and feasibility of the intervention, and specific services, including MH. Interviews were audio recorded, transcribed verbatim, and then translated into English where necessary. Written informed consent was obtained before the interviews were initiated and pseudonyms were used throughout for confidentiality and anonymity.

### Qualitative data analysis

For this paper, qualitative data that related specifically to the acceptability of integrated services and, more specifically, the MH service were identified and selected from a larger qualitative dataset. This included non-participant observation notes, three FGDs with healthcare providers, and three FGDs and five IDIs with clients. From familiarisation of the data, qualitative analytical discussion between CMY and MT, and discussion of quantitative findings, themes were identified. Data were coded in Nvivo 12. Analytical memos were written to analyze, and organize emerging findings, and as a basis for iterative analytical discussion [34]. This approach allowed for the generation of unanticipated insights and a nuanced understanding of participants’ experiences with CHIEDZA and the MH service within it. Non-participant observations of CHIEDZA sites and observations of monthly study team meetings primarily aimed to understand intervention implementation, adaptations, and context.

Other papers detailing qualitative findings of the CHIEDZA intervention have been published elsewhere [25, 35].

## Results

Overall, 75.0% (27,725/36991) of the clients that accessed CHIEDZA services between April 2019 – March 2022 were female. Over half of these female clients (16,600, 59.9%) only visited the CHIEDZA sites once and this was similar when across provinces (Harare 59.8%; Bulawayo 54.6%; Mashonaland East 64.5%). Of the remaining 40.1% of female clients, 5353 (19.3%) accessed CHIEDZA services twice, 2254 (8.1%) three times, 1209 (4.4%) four times, and 2039 (8.3%) five or more times.

### Uptake of menstrual health services

**Fig. 1:**
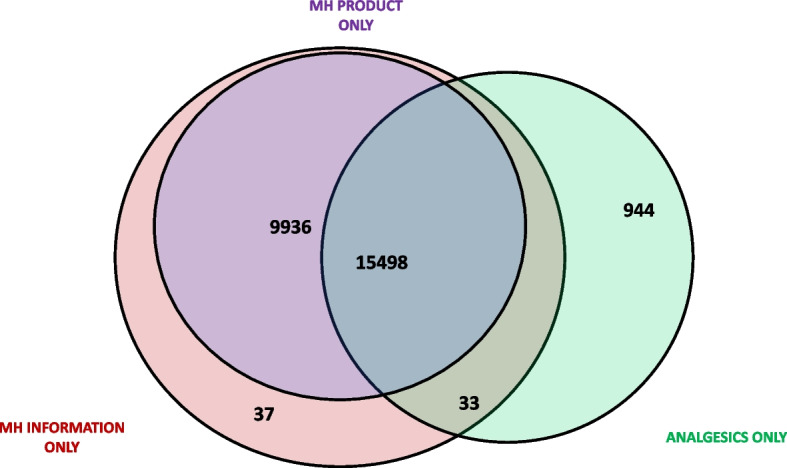
Uptake of MH service disaggregated by MH service components

Almost all female attendees (*n* = 26,448; 95.4%) accepted an MH service, of whom 58.6% (*N* = 15,498) took MH information, MH product, and analgesics, 37.6% (*N* = 9936) took MH information and MH product, 3.6% (*N* = 944) took MH analgesics only, 0.1% (*N* = 37) took MH information only, and 0.1% (*N* = 33) took MH information and MH analgesics only (Fig. 1).

Overall, MH service uptake was consistently high with over 85% uptake per visit throughout the intervention period. However, there were periods between July – March 2020 that showed a decrease in MH service uptake with 85.8 and 87.3% uptake from July – September 2020 in Harare and Mashonaland East respectively and 85.4 and 79.3% uptake from October – December 2020 uptake from Harare and Mashonaland East respectively.

Most CHIEDZA clients (24,029; 86.7%) took up MH services at their first visit. Of those who did not take up MH services at the first visit, 37.3% took up MH services at the second visit, and 19.1% took up MH services on a subsequent visit, with a similar pattern across the three provinces.

### Uptake of analgesics

During the intervention timeframe, 59.4% (16,475/27725) of female CHIEDZA clients took analgesics (ibuprofen and/or paracetamol). Uptake was highest in Bulawayo (6670/8404; 79.4%) compared to Harare (5340/9612; 55.6%) and Mashonaland East (4465/9709; 46.0%). In terms of analgesic choice, most clients only took up ibuprofen (8332/15475; 50.6%) as opposed to only paracetamol (5817/16475; 35.3%), and some clients took both (2326/16475; 14.1%). In Harare, ibuprofen was taken more than twice as often as paracetamol. In Bulawayo and Mashonaland East, ibuprofen and paracetamol had similar uptake.

Most participants only took up analgesics at one visit (12,011/16475; 79.2%). However, one participant took up analgesics at each visit for up to 24 visits. The median time between any two visits was 80 days (IQR: 52–98 days).

### Factors that informed MH service uptake

Qualitative findings from interviews with CHIEDZA providers and clients provided a nuanced understanding of the factors that informed uptake of the MH service. For example, when asked what young people appreciated about CHIEDZA, a client noted that they appreciated the provision of free services, specifically MH products, that they could no longer afford:

“*CHIEDZA helped a lot of young people by giving us free services. Condoms are expensive to buy these days, pads are expensive too so these free services were really helpful…*” (Client IDI, male, 25 years old).

Clients also appreciated the youth-friendly and non-judgemental service delivery offered at CHIEDZA and many felt very comfortable coming to CHIEDZA to not only receive free MH products, but also to talk about how to use the MH products and about menstruation and SRH more generally:

*“When I came to CHIEDZA I saw a youth worker who wasn’t shy at all. He really explained that a pad works in such and such a manner, condoms are supposed to be worn like this and this and it came to my mind that there really isn’t anything embarrassing after all. I… actually realized that there is nothing to be ashamed of.”* (Client IDI, female, 19 years old).

Qualitative findings also highlighted the prevalence of MH-related pain and the importance of analgesic provision from CHIEDZA as clients described how menstrual pain limited their mobility and often affected their ability to engage in social and economic activities. When asked if one could go to work or church when experiencing menstrual pain, clients responded:

*“It is impossible… You will do nothing with that pain,”* (Client IDI, Female, 22 years old).

*“I usually have a massive period pain and so I stay at home most of the times. The fact that I stay at home for two or four days affects my routine and practices because am a dancer I do not practice until I finish,”* (FGD, Female, 16–19 years old).

When discussing how they managed MH-related pain, many clients noted that they used free analgesics from CHIEDZA or bought analgesics from the pharmacy when they can afford them. Other pain management options used included “*drinking hot water*” or “*using heat*”.

### Menstrual health product choice

**Fig. 2:**
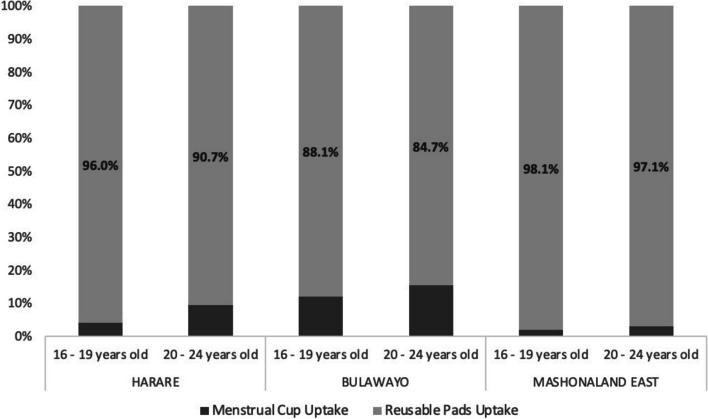
MH product choice stratified by province and age group

Of the 25,433 clients that took up MH products from April 2019 – March 2022, most chose reusable pads (23,613/25433 (92.8%). There was strong evidence of a difference in product choice by age with 778/13136 (5.9%) of 16–19 year olds compared to 1042/12297 (8.5%) of 20–24 year olds choosing menstrual cups as opposed to reusable pads (Fig. 2). There was also strong evidence of a difference in MH product choice by province with a higher proportion of clients choosing the menstrual cup in Bulawayo (1023/7638, 13.4%) as opposed to Harare (587/8832, 6.7%) or Mashonaland East (210/8963,2.3%). In a model with a random effect for cluster, selecting the cup was associated with being aged 20–24 (aOR = 1.65, 95%CI 1.49–1.82, *p* < 0.001), and residing in Harare (aOR 3.03, 95% CI 2.08–4.41) or Bulawayo (aOR 6.87, 95%CI 4.73–9.96).

Of the clients who took up an MH product, 386/25433 (1.52%) clients chose to swap MH products at least once during the intervention timeframe with 303/1820 (16.7%) swapping from the menstrual cup to reusable pads but only 83/23613 (0.4%) swapped to the menstrual cup (Table 1). Discontinuation of the menstrual cup in favour of reusable pads varied by province, with higher discontinuation in Bulawayo (192/1023; 18.8%) compared to Harare (77/587; 13.1%) or Mashonaland East (34/210; 16.2%). Discontinuation of the menstrual cup in favour of reusable pads also varied by age group, with higher discontinuation among 16–19 year olds (158/778; 20.3%) compared to 20–24 year olds (145/1042; 13.9%). In a model with a random effect for cluster, swapping from menstrual cup to pads was associated with higher odds of being aged 18–20 (aOR = 1.49, 95%CI 1.16–1.92) but there was no association with province.

**Table 1:** MH product choice and continued use vs. discontinued use by province

	Initial MH Product Choice	Age Group (years old)	N	Ever swapped to other MH product n (%)
**TOTAL**	Menstrual Cup	16–19	778	158 (20.3%)
		20–24	1042	145 (13.9%)
		**TOTAL**	**1820**	**303 (16.7%)**
	Reusable Pads	16–19	12,358	34(0.3%)
		20–24	11,255	49 (0.4%)
		**TOTAL**	**23,613**	**83 (0.4%)**
**HARARE**	Menstrual Cup	16–19	179	30 (16.8%)
		20–24	408	47 (11.5%)
		**TOTAL**	**587**	**77 (13.1%)**
	Reusable Pads	16–19	4264	13 (0.3%)
		20–24	3981	25 (0.6%)
		**TOTAL**	**8245**	**38 (0.5%)**
**BULAWAYO**	Menstrual Cup	16–19	518	116 (22.4%)
		20–24	505	76 (15.0%)
		**TOTAL**	**1023**	**192 (18.8%)**
	Reusable Pads	16–19	3827	16 (0.4%)
		20–24	2788	19 (0.7%)
		**TOTAL**	**6615**	**35(0.5%)**
**MASH EAST**	Menstrual Cup	16–19	81	12 (14.8%)
		20–24	129	22 (17.1%)
		**TOTAL**	**210**	**34 (16.2%)**
	Reusable Pads	16–19	4267	5 (0.1%)
		20–24	4486	5 (0.1%)
		**TOTAL**	**8753**	**10 (0.1%)**

The median time between first MH product choice and swapping to second MH product choice was 343 days (IQR 133–490 days).

Most clients (365/386; 94.6%) that swapped did so only once. However, 18 clients swapped products twice (16 reusable pads – menstrual cup – reusable pads; 2 menstrual cup - reusable pads - menstrual cup), one participant swapped three times (menstrual cup - reusable pads - menstrual cup – reusable pads), and two swapped four times (menstrual cup - reusable pads - menstrual cup – reusable pads). Among the 23 participants who swapped more than once, the median time from the first product choice to first swap was 224 days (IQR 140–378, range 7–476) and the median time from then to the second switch was 175 days (IQR 140–413).

After 12 months or more, 2969 and 367 clients swapped their old reusable pads for new reusable pads once or more than once respectively. This was done as old reusable pads were no longer sufficiently absorbent. The median time between swapping of old reusables for new ones was 399 days (IQR: 343–490 days).

Interviews with CHIEDZA clients highlighted the factors that informed product choice. Some clients chose the menstrual cups either because they had a heavy flow and felt that the cup would be less likely to leak or because they were uncomfortable with the idea of carrying around a wet reusable pad or washing and drying their used reusable pads:

*“Well… I took a cup and I am very comfortable with my cup as I hate washing pads with blood. I can wear my tight jeans and there are no markings or signs that I am wearing it unlike when wearing a pad,”* (Client IDI, Female, 20 years old).

*“Many indeed are not comfortable with the act of washing a reusable pad… That’s why some wanted to adopt the cup. They take the cup because they do not want to look at their blood and worse still wash it,”* (CHIEDZA service provider IDI, Bulawayo).

However, most clients, particularly younger women, were uncomfortable with the use of insertable MH products such as the menstrual cup. Clients either feared that it would be painful “*down there*” or believed cup use contradicted longstanding beliefs around “virginity” and the importance of preserving the hymen and keeping it intact before marriage. This barrier to insertable menstrual cups due to “virginity” or fear was highlighted when clients were asked about product choice and swapping:

*“I chose the pads and if the pads were not there, I was not going to choose the cup because I have never used a cup and I am scared to put things down there.”* (Client FGD, Female, 16–19 years old).

*“When I came [to CHIEDZA] they did not have pads so I took a cup… It was not a comfortable experience for me because I am still young and so I returned it after 3 months…”* (Client FGD, Female, 16–19 years old).

*“The pads are not difficult to wash and they are also very comfortable… Most people are scared of the cup because they think it will affect their virginity,”* (Client FGD, Female, 16–19 years old).

#### Stock-outs

MH product stock-outs during the CHIEDZA implementation period occurred mostly between July – December 2020 (data available in supplementary material). Overall, CHIEDZA sites in Bulawayo were particularly affected with 1164 incidences as opposed to 662 and 646 in Harare and Mashonaland East respectively. Interviews with providers highlighted that high stock-outs in Bulawayo were due to logistical delays in getting products from Harare to Bulawayo.

#### Other services within CHIEDZA

**Fig. 3:**
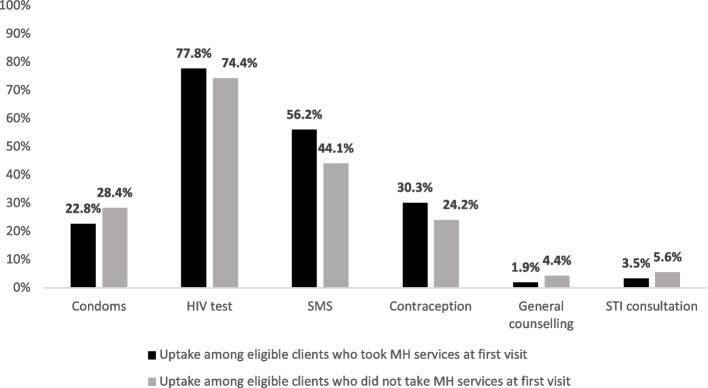
CHIEDZA services uptake among female clients who took up MH services at first visit vs those who did not take up MH services at first visit

Overall, the MH services had the highest uptake at first visit (95.5%) for female clients. Of these, 1747 clients only took up the MH services at first visit and the rest took up MH services and other services such as HIV testing (77.8%), SRH sensitization via SMS (56.2%), and contraception (30.3%) (Fig. 3).

Only 4.9% (1359/27725) of clients did not take up the MH service at first visit and 8.1% (2254/27725) found their MH product of choice was out of stock at first visit.

For female CHIEDZA clients that visited CHIEDZA more than once and were eligible to receive other services at those visits, most took up the MH services, HIV testing, SRH sensitization via SMS, and family planning:

*“The first time she visited the site, she self-tested for HIV, received sanitary pads, pants, pain killer tablets for period pains and counselling services on sexual relationship…”* (Client, IDI, 18 years old).

The pathways through which the integrated nature of the CHIEDZA intervention positively informed client engagement with MH and other SRH services was especially highlighted during interviews with CHIEDZA service providers and clients. Providers described how both the integration of services in CHIEDZA and the popularity of the MH service among young women in the communities led to clients coming to CHIEDZA for MH products and then being exposed to or taking up more services such as HIV testing:

“*Initially the client thought that CHIEDZA is a program which offers pads only but when she got there, she was surprised to hear that a range of services are in store*,” (CHIEDZA service provider IDI, Male).

*“She indicated that she was invited by her friend to get pads at CHIEDZA and that is how she later got tested, HIV positive.”* (Client IDI, Female, 22 years old).

Client interviews also revealed that the popularity of the MH services within CHIEDZA facilitated an outward-facing branding of the intervention that did not focus SRH thus making it easier for young people, particularly young women, to visit CHIEDZA without fear of judgement from peers and the larger community. This feeling of ease and comfort with engaging with CHIEDZA was shown in how young people chose to come with family or groups of friends and did not shy away when they were noticed by other members of the community:

“*Young people do come to CHIEDZA as groups of friends; two girls walked into the community centre together and noticed their friend… and they all waved to each other in greeting*.” (Non-participant site observation).

## Discussion

This study shows a very high uptake of MH services offered as part of integrated HIV and SRH services. The MH services were the most accessed CHIEDZA services among young women across all provinces. This reflects a large unmet need for a choice of MH products, analgesics, and education among young women across Zimbabwe and adds to the evidence base for the need for comprehensive MH interventions in community-based settings [16, 29, 36].

The study demonstrates that the integration of MH in community-based SRH interventions may be an effective implementation pathway to increase female engagement and uptake of other critical SRH services such as HIV testing or family planning. It demonstrates the value of integrated service provision that places MH at the centre of SRH service provision for women [18, 36, 37]. When provided with a range of SRH services within an integrated package, young women, regardless of age or geographical location almost always chose to take up MH services. Given these findings, it is essential that MH is integrated in SRH interventions for young women. Alongside the MH services, young women took up HIV testing, SRH sensitization, analgesics, and condoms at first and subsequent visits, suggesting these additional services are important to young women and thus should be prioritized in integrated SRH service provision. Moreover, these MH services may act as “hook”- encouraging the uptake of services such HIV testing and/or contraception that may need more time and understanding before being taken up [25, 38, 39].

Most current MH interventions provide access to either reusable pads or menstrual cups but not both [30, 40]. Our findings suggest that factors such as age, geographical location, and sociocultural norms inform MH product choice and use. Women, particularly younger women, in Zimbabwe preferred reusable pads to the menstrual cup and the uptake and acceptability of the cup was low [30]. Of the few young women that did opt for menstrual cups, many later swapped out the menstrual cup for reusable pads. However, very few clients chose to swap out the reusable pads for the menstrual cup in the study. In contrast to other studies [41, 42], we found that, given product choice, menstrual cups were not the preferred MH product among young women in Zimbabwe. The reluctance to opt for and/or use the menstrual cup may be due to an unwillingness to try a novel menstrual product or unwillingness to use an insertable product that may affect their “virginity” [28, 30, 43]. These barriers persisted despite MH and menstrual cup sensitization and education provided through group talks, educational materials, and “menstrual cup champions” on site [44].

A closer look at the product choice and patterns of use in Bulawayo also provide insight into the importance of product choice. Bulawayo had the highest menstrual cup uptake and highest product swapping from menstrual cups to reusable pads. We hypothesise that in the absence of product choice (when reusable pads were not available), young women chose to take up the menstrual cup, but when given the opportunity to swap after 3 months, many opted to swap the menstrual cup for reusable pads once available. While young women Bulawayo were more willing to try novel products such as the menstrual cup, their preference over time was reusable pads. This preference may be informed by user experience or other factors but it is important that interventions address this change by providing informed choice when addressing unmet MH needs [2, 40].

The overall low incidence of MH product swapping itself highlights that when given the information and support needed to make an informed decision regarding MH product choice, most young women are happy with their initial choice. Youth-friendly and non-judgemental MH service delivery allows for young women to feel comfortable enough to come back to service for more guidance on MH product use and/or guidance on other options to manage their menstruation.

As well as provision of products, analgesia is a critical component of MH services. There is limited research on MH-related pain among young women, particularly in LMICs [45]. Other studies including a systematic review of studies investigating menstrual experiences in high income countries (HICs) [46], show that menstrual pain is associated with lower engagement in work and school. We found high uptake of analgesics with a significant proportion of clients returning repeatedly for analgesia, with ibuprofen being the preferred over paracetamol. MH interventions should include pain management through the provision of a choice of analgesics or other methods such as hot water bottles.

As reported in other studies of many health interventions, more females than males engaged with the CHIEDZA service. The high MH service uptake at first visit and the popularity of the MH service overall suggests that the MH service may have had a particularly strong influence on bringing young women to CHIEDZA. This finding is further supported by a qualitative study we conducted to explore the acceptability of the MH service within CHIEDZA [44]. Here, particularly during the COVID-19 pandemic, when markets were closed, less money was available in the household, and social activities and games within CHIEDZA were suspended, the MH service became the sole “*pull factor*” framing CHIEDZA as a “women’s service” and encouraging young women to visit CHIEDZA [44, 47]. These findings are similar to those found in a study looking at MH access through a peer-led SRH service for youth in Zambia before and during the COVID-19 pandemic [13]. In the Zambian study, access to MH products and education through community-based SRH service addressed an unmet need for MH support and proved to be essential to young women in the community, particularly during the COVID-19 pandemic [13].

External factors such as supply chains inform service implementation and delivery [48]. In our study, dips in MH service uptake coincided with recorded incidences of stock-outs and the COVID-19 restrictions in Zimbabwe in 2020. In March of 2020, the Zimbabwean government announced a mandatory nationwide lockdown [49] and all CHIEDZA service provision was paused for 6 weeks from March 30th – May 18th, 2020. This lockdown also negatively affected procurement and delivery of MH products to CHIEDZA sites across the country, highlighting the importance of consistent MH product supply in MH and SRH service delivery.

To our knowledge, this is the first study to investigate provision of MH services in SRH services. Our MH service included education, menstrual products, and analgesics, with choices of products and analgesics provided [18, 45]. We were able to track MH service uptake as well as uptake of other services at individual level over time [40]. The study had a large sample size and was conducted across three provinces in Zimbabwe, including the two main ethnic groups.

Our study also had some limitations. The MH service did not include other forms of MH pain management. However, information on other forms of pain management was disseminated on site. We also did not collect detailed quantitative beyond age, sex, and provincial location but this was done for a MH cohort study detailed elsewhere [31]. Additionally, observed uptake of the MH products may not have equated to use over time. However, this was further explored qualitatively in another study that showed high uptake was also linked to high use of MH products, particularly the reusable pads [44].

## Conclusion

Overall, the study results showed high uptake of the MH service among young women as part of a multicomponent SRH service package. The provision of MH education, analgesics, and a choice of MH products through comprehensive MH interventions may serve as a pathway to increase female engagement with other important SRH services such as family planning and HIV testing [17, 18, 44, 50]. These study findings should encourage policymakers to increase their commitment to integrated service SRH provision that sets MH as a key part of reproductive health for young women.

### Supplementary Information

**Supplementary Material 1.**

## Data Availability

The datasets used and/or analysed during the current study are available from the corresponding author on request.

## Abbreviations

CHW: Community health worker
FGD: Focus group discussion
IDI: In-depth interview
LMIC: Low- and middle-income countries
MH: Menstrual health
MoHCC: Ministry of health and child care
RA: Research assistant
SDGs: Sustainable development goals
SRH: Sexual and reproductive health
UNICEF: United nations children’s fund
WASH: Water, sanitation, and hygiene
WHO: World health organization
