# Supplementary material for: High uptake of menstrual health information, products and analgesics within an integrated sexual reproductive health service for young people in Zimbabwe
Source: Reprod Health. 2024 Apr 22;21:56. doi: 10.1186/s12978-024-01789-y (PMC11036648; doi:10.1186/s12978-024-01789-y)
Supplement: Supplementary file 1 — Supplementary Material 1. [file 12978_2024_1789_MOESM1_ESM.pdf]

Table 1. Recorded incidences of MH product stock-outs

	2019			2020				2021					
	Apr-Jun	Jul-Sep	Oct-Dec	Jan-Mar	Apr-Jun	Jul-Sep	Oct-Dec	Jan-Mar	Apr-Jun	Jul-Sep	Oct - Dec	Jan - Mar	TOTAL
HARARE	56	138	0	0	28	289	83	2	1	65	-	-	662
BULAWAYO	-	58	13	258	165	355	236	0	1	65	13	-	1164
MASH EAST	-	0	0	0	0	277	312	2	2	48	0	-	646
